# Comprehensive study on impact of hydrogen peroxide decomposition on the crucial parameters of OSM-type energetic materials

**DOI:** 10.1038/s41598-024-64974-w

**Published:** 2024-06-18

**Authors:** Mateusz Polis, Agnieszka Stolarczyk, Konrad Szydło, Magdalena Fabin, Mateusz Pytlik, Barbara Lisiecka, Tomasz Jarosz

**Affiliations:** 1 Department of Physical Chemistry and Technology of Polymers, Silesian University of Technology, 44-100 Gliwice, Poland; 2 Explosive Techniques Research Group, Łukasiewicz Research Network - Institute of Industrial Organic Chemistry, Krupski Młyn, 42-693 Poland; 3 National Research Institute, Conformity Assessment Body, Central Mining Institute, 1 Gwarków Square, 40-166 Katowice, Poland

**Keywords:** Chemistry, Materials science

## Abstract

The use of increasingly advanced energetic materials (EMs) in various branches of industry and military sectors increases the appropriate requirements for EMs, including: their durability, safety of use, chemical and high-energetic properties. Additionally, the impact of the products of the explosion of EMs on the natural environment is also crucial. Therefore, on-site mixture (OSM) energetic materials containing concentrated hydrogen peroxide (OSM-type energetic materials) are becoming increasingly popular. This is an extremely interesting group of materials that contains in excess of 50 wt.% hydrogen peroxide (HP) and not containing toxic compounds, and therefore is environmentally friendly. The main objective of the study was to investigate the various compositions of OSM-type energetic materials in terms of the evolution over time of their energetic properties (including the “raw” energetic material strength and the ability to sustain the propagation of a detonation wave) and the volume of the post-detonation gases. The obtained results show that the decomposition of hydrogen peroxide strongly affects the detonation parameters of OSM-type energetic material and the decomposition time of HP. In addition, it has been proven that rate of decomposition of HP significantly affects the detonation parameters of OSM-type energetic materials. It was also found that the concentration of NO$_{\hbox {x}}$ is low and decreases dramatically with the decomposition of hydrogen peroxide, but at the same time the concentration of carbon oxides increases.

## Introduction

Energetic materials (EMs) have found an extensive variety of civilian (mining, demolitions, civil engineering) and military applications^1,2^, with civilian applications making up the majority of the annual usage of EMs. The main groups of EMs utilised in civilian applications are ammonium nitrate-based and nitric acid ester-based EMs. The performance of EMs is typically compared based on velocity of detonation (VoD) parameters, with ammonium nitrate-based EMs typically exhibiting low performance (VoD in the range of 2000 – 4000 m/s) and nitric acid ester-based EMs exhibiting moderately high performance (VoD on the order of 5000 m/s).

The manufacture, storage and use of EMs, however, is associated with chemicals that are hazardous to human health or have a significant adverse effect on the environment. Such substances may be contained in EM formulations as their components (e.g. nitrocompounds)^3–6^, released from EMs during storage (e.g. precipitation of nitric acid esters from dynamites) or produced upon detonation (e.g. carbon monoxide, nitrogen oxides present in post-detonation gases)^7^. Moreover, in the case of non-ideal detonation or deflagration of the EM inside a borehole, the environment can be contaminated with both the constituents of an EM and with its decomposition products. Examples of the former include nitric acid esters (in the case of dynamites), nitrates, sodium thioacetate, thiourea or sodium perchlorate (in the case of emulsion explosives).

Among the problematic substances associated with EMs, nitric acid esters that exhibit explosive properties are particularly prominent, as they are widely used in the energetic materials industry. This group is best exemplified by substances, such as propane-1,2,3-triol trinitrate (commonly referred to as “nitroglycerine”, NG), ethane-1,2-diol dinitrate (commonly referred to as “nitroglycol”, NGC), pentaerythritol tetranitrate (commonly referred to as “penthrite”, PETN) and cellulose nitrate (commonly referred to as “nitrocellulose”, NC). These substances are used both as independent explosives (e.g. the use of PETN in detonators as the main source of the shock wave)^8,9^ and as components of the formulations of secondary explosives (e.g. the use of NG and NGC in dynamites)^10^, propellants (e.g. the use of NG and NC in double-base gunpowders)^11,12^ and pyrotechnics (e.g. the use of NC as a binder)^13^.

The widespread use of those substances in a variety of technologies has a significant impact on both the environment and on personnel health. In terms of their impact on the former, the most common nitric acid esters exhibit the following adverse effects:

- Pentaerythritol tetranitrate: acute toxicity to crustaceans (Daphnia manga)^14^, sublethal effect: invertebrates (Eisenia andrei) and plants (Lolium perrenne)^15^;
- Propane-1,2,3-triol trinitrate: acute toxicity to plants (Medicago sativa, Echinochloa crusgalli, Lolium perenne) and bioaccumulation in the form of pentaerythritol dinitrate^16^;
- Ethane-1,2-diol dinitrate: acute toxicity to plants (Sinapis alba, Juncus inflexus, Phragmites australis, Linum usitatissimum)^17^.

The impact of nitric acid esters on human health is commonly thought of as positive, because both NG and PETN in low doses are used to treat the symptoms of coronary heart disease^18,19^. Recent studies, however, indicate that long-term therapeutic use of these compounds may have a toxic effect, inducing, among others, endothelial dysfunction^20^. There are also reports that NG accelerates the regeneration of tissue damage caused by exposure to thioacetamide. In larger doses^3^ or through chronic exposure^4^, however, these compounds are harmful or even toxic^5,6^.

The risk of exposure to nitric acid esters used in the EM industry is only exacerbated by the fact that liquid nitric acid esters (i.e. NG or NGC) are known to migrate from the bulk of the EM towards its surface^21,22^. Migration is followed by precipitation of the liquid nitric acid esters on the surface of the EM. This presents not only a significant threat in terms of chemical contamination of the environment and personnel exposure to these substances, but also in terms of the risk of an accidental explosion.

These issues have sparked significant interest in “green” EMs that would avoid such drawbacks. An important development in this area of study was the use of concentrated hydrogen peroxide (i.e. aqueous solutions containing >50 wt. % hydrogen peroxide, HP) as an oxidising agent. HP is currently most often produced in the anthraquinone process, in which the raw materials are hydrogen and air, and anthraquinone is the mediator, which is used in a closed cycle. This process has only minimal impact on the environment and according to literature reports, work on a modification of this process, which would be completely neutral to the environment, is ongoing^23^. Due to its high chemical reactivity and easy decomposition into oxygen and water, HP cannot contaminate the environment or bioaccumulate. Moreover, concentrated HP can undergo detonation and achieve VoD values of up to 6600 m$\cdot$s^−1^. However, the high critical diameter (40.6 mm at 50 $^{\circ }$C) of HP solutions makes their direct use impractical^24^.

The minimal environmental footprint of HP makes it an oxidising agent that is attractive from the viewpoint of minimising the environmental impact of EMs, especially in comparison to substances present in classical EMs. Due to this, several works have explored the possibility of producing HP-based EMs, primarily in the form of mixtures with liquid organic fuels, such as propane-1,2,3-triol, acetone and ethanol^25,26^. However, the amount of information available regarding this type of explosives is limited due to the minimal use of liquid explosives in blasting.

Further works on HP-based EMs typically concern systems containing viscosity modifiers or gelling agents^27^. These substances prevent the flow of explosives containing concentrated HP, reducing the risk associated with their leakage, reducing the available surface area of contact between concentrated HP and potential contaminants, and enabling the loading of these explosives into blasting holes with different spatial orientation. The above modifications contributed to the renewal of research interest in this group of EMs, in the context of environmentally friendly explosives formulas, and in particular due to the possibility of developing systems that will not emit nitrogen oxides as a result of detonation^28,29^. This renewal has resulted in the development of on-site mixed (OSM-type) EMs, which are characterised by the fact that they can be produced on the site of planned blasting operations, from non-explosive ingredients, as in the case of bulk emulsion EMs. These OSM-type EMs are of particular interest, as they contain no nitric acid esters or other highly problematic compounds^28,30^. Simultaneously, this group of EMs either marginal amounts of nitrogen-bearing compounds (typically in the form of nitrates) or does not contain such compounds at all, leading to limited emission of nitrogen oxides (NO$_{\hbox {x}}$) upon detonation^29^.

In terms of energetic performance, OSM-type EMs typically show relatively high detonation parameters^28,30,31^ eg. high detonation velocities (referred in Supplementary Material), which are comparable to those typical of dynamites^32^ and achievable by special-purpose EMs, but significantly exceeding the parameters typical of ammonium-nitrate EMs^33,34^. The achievable detonation parameters are largely dependent on the selection of fuel, gelling agent and auxiliary substances used. Literature also reports that OSM-type EMs were initiated almost without failure, indicating their low susceptibility to the phenomenon of interrupted detonation^28,35^.

An important limitation of this group of EMs, however, is the fact that HP is inherently highly reactive. Although high-purity HP solutions of even very high concentration (i.e. >90 wt. %) are relatively stable (loss of approx. 1 wt. % H$_{\hbox {2}}$O$_{\hbox {2}}$ when stored at 30 $^{\circ }$C over the course of a year), the introduction of any impurities drastically decreases that stability^25^. Due to the fact that many of the components of such energetic formulations can promote the spontaneous decomposition of HP, this process needs to be taken into consideration when producing and utilising OSM-type EMs. That said, the “built-in” decomposition of HP in these EMs can also be utilised as a safety feature. In the case of theft or misfires, once the concentration of HP falls below a certain point, the EM is unable to sustain detonation, preventing its criminal misuse or mitigating the threat of undetonated EM being left in the blasting holes after a misfire. For this safety feature to be effective, the rate of HP decay needs to be strictly controlled, i.e. delays in blasting operations (typically up to 4 – 5 h) should not result in a loss of EM performance, but in the case of a misfire, the deactivation of the EM should not cause undue delays in blasting operations. As such, an optimal lifetime of the investigated OSM-type EMs would be in the range of 24 – 48 hours.

Despite the number of works on OSM-type EMs, the issue of HP decomposition in these EM formulations has only been investigated in our previous works^31,36^. In those works, we have focused first on the impact of the addition of Al powder and later on the impact of the choice of auxiliary oxidising agents, gelling agents and even physical sensitising agents on the decomposition of HP. That said, no concerted and comprehensive research efforts have thus far been undertaken to connect the changes in the HP concentration, taking place over time, with the changes in the energetic parameters of the OSM-type EMs.

Consequently, in this work we have investigated a series of OSM formulations in regards to the evolution of their energetic properties over time. This included both the “raw” EM strength expressed by the velocity of detonation (VoD), brisance or air blast parameters, as well as the ability of the OSM-type EMs to sustain the propagation of a detonation wave, expressed by the critical diameter of the formulations. Corroborating these results with those presented in our previous work reveals a clear picture of the impact of HP decomposition on the energetic performance of OSM-type EMs. Counter-intuitively, this connection and evolution over time is not monotonous, but reveals a competition between the decay of the main energetic component (i.e. HP) and the increasing population of hot-spots caused by the evolution of oxygen bubbles produced through this decay.

The other key aspect of this work was that we have for the first time investigated the environmental impact of OSM-type EMs by examining the composition of post-detonation gases and quantifying the amounts of emitted toxic products.

## Materials and methods

### Preparation of on-site mixture samples

The preparation of OSM samples, i.e. OSM-Ca, OSM-K and OSM-Na, has been conducted in a DRAIS-type mixing unit. First, the auxiliary oxidising agent Ca(NO$_{\hbox {3}})_{\hbox {2}}$ $\times$ 4H$_{\hbox {2}}$O, KNO$_{\hbox {3}}$ and NaNO$_{\hbox {3}}$, respectively for OSM-Ca, OSM-K and OSM-Na was pre-mixed with glycerine over a period of 5 minutes (100 RPM). Next, the calculated amount of hydrogen peroxide solution was added into mixing vessel and the mixing process has been continued for 10 minutes. Next, the guar gum (binder) was added and mixing was conducted until full homogenisation of the mixture. Lastly, glass microspheres were gently added and mixed at a lower setting (20 RPM) to minimise both the aeration of the composition and microsphere breakage. The microspheres added to EM formulations play the role of hot-spots, which facilitate the development of detonation^37^. The mixing process was performed for 1.5 kg batches. The ageing time was calculated since hydrogen peroxide solution was added into the chamber of the mixing unit. The composition of the investigated samples is presented in Table 1. The reagents and chemicals used in this work are listed in Supplementary Material Table A1. All chemicals were used without purification.

**Table 1:** Composition of samples investigated in this work, given in wt. %.

Component	OSM-Ca	OSM-K	OSM-Na
NaNO$_{\hbox {3}}$	–	–	9.9
KNO$_{\hbox {3}}$	–	9.9	–
Ca(NO$_{\hbox {3}}$)$_{\hbox {2}}$ $\times$ 4H$_{\hbox {2}}$O	9.9	–	–
HP	71	71	71
Glycerine	15.1	15.1	15.1
GG	3	3	3
MS	1	1	1

### Charge preparation and ageing

The produced OSM samples were loaded into prepared polypropylene tubes (l = 250 mm, internal diameter = 46.4 mm, wall thickness = 1.8 mm). The tubes were sealed at one end. Inside the tube, transverse to the axis of the charge, four short-circuit probes were placed, so as to allow VoD to be measured via the discrete method. The first probe was placed 120 mm from the initiation point and subsequent probes were placed every 40 mm after this probe. After the tubes were loaded, the charges were weighed, tightly closed and equipped with 16 ± 0.2 g boosters, made of AIX-1 EM (95 wt. % RDX, 5 wt. % wax). The boosters were placed axially on one end of each charge. Each of the used boosters was cylindrical in shape (d = 21 ± 0.02 mm, h = 30 ± 0.02 mm), with a detonator cavity (d = 8 ± 0.05 mm, h = 18 ± 0.05 mm) located in the axis of the booster. Boosters were prepared via pressing under 4 MPa. The charges were initiated using NITRODET 0.2 A detonators (produced by Nitroerg S. A., Bieruń, Poland). The conical charges for critical diameter measurements were prepared in a analogous sequence of unit operations.

The ageing of the OSM samples was conducted for loaded charges, so as to avoid handling the partially decomposed OSM samples. Ageing was conducted in a closed chamber, at a temperature of 20 $^{\circ }$C and relative humidity of 50 %. After a set time (1, 3, 6 or 24 hours) had elapsed, each set of charges was taken out of the chamber and investigated, as detailed in the manuscript.

### Velocity of detonation and pressure parameters measurement

**Figure 1:**
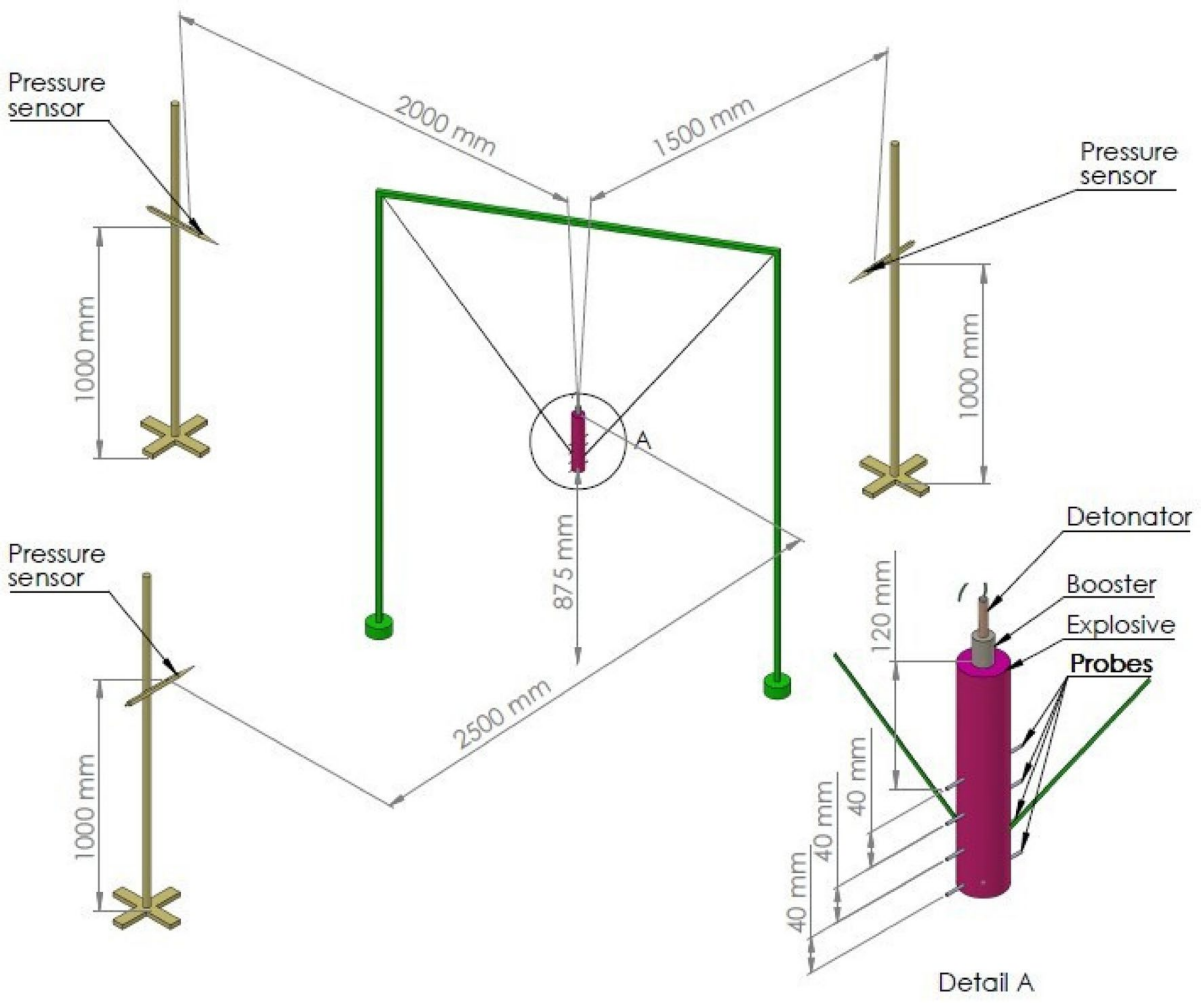
Set-up for simultaneous pressure parameters and detonation velocity testing.

The tested charges were suspended 1 m above the ground level, measured from the middle of charge. Three piezoelectric pressure sensors (137A23 and 137A22 produced by PCV Piezotronics) were placed 1.5; 2 and 2.5 m from the charge axis and 1 m above the ground level (Fig. 1). Pressure sensor data were recorded using a AMC VIBRO CONDITION 8000D signal conditioner and Tektronix TBS2401B digital oscilloscope (Figure A1).

The accuracy of the measurement was limited primarily by the sensitivity of the pressure sensors (1.42 mV/Pa ± 15%, 2.692 mV/Pa ± 15% and 2.768 mV/Pa ± 15%) and sampling rate of the oscilloscope (10 MSa/s). The velocity of detonation was recorded for approx. 450 g charges simultaneously with pressure sensor data, using short-circuit probes that the charges were equipped with. VoD data was recorded using the AMC VIBRO CONDITION 8000D signal conditioner and a Rigol MS05104 digital oscilloscope. The accuracy of the measurement was limited primarily by the precision, with which the probes could be assembled (±1 mm). The velocity of detonation was calculated based on the time intervals between the short-circuiting of the probes.

### Brisance

**Figure 2:**
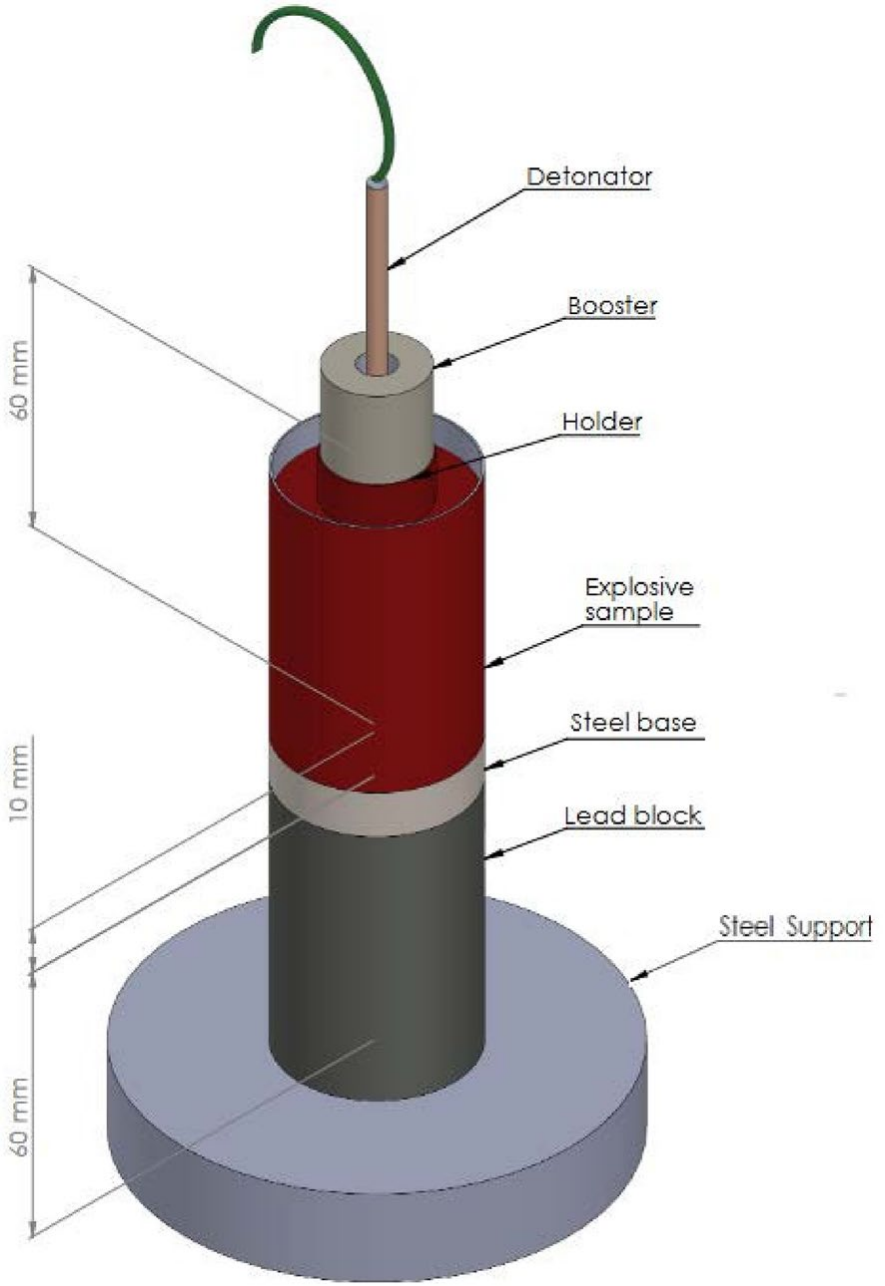
Simplified scheme of the experimental set-up used for measuring brisance.

The brisance of the OSM smaples was invesitgated via the modified Hess lead block compression test (Fig. 2)^38^.

The cylindrical charge casings (d = 40 mm, h = 60 mm, wall thickness = 0.5 mm) were 3D printed (Prusa Mk3) from PETG polymer (Prusament PETG PRUSA Filament). Approx. 50 g of the tested OSM sample was loaded into each casing. Next, each casing was covered with a fitted enclosure that was equipped with a central cavity for inserting a booster and detonator. The charges were initiated identically as in the VoD and air blast pressure measurements. The boosters were used in order to maintain compliance with the other conducted tests. Class 5 RDX (Nitrochem S. A., Bydgoszcz, Poland), pressed to 1 g/cm^3^ and initiated in the same manner was used as the reference explosive.

Lead cylinders (99.997% pure lead, d = 40±0.2 mm, h = 60±0.15 mm) were used for this measurement. The height of all cylinders was measured in four points prior to the brisance measurement. For the measurement, the cylinder was placed on a steel support plate and covered with a steel ring (d = 41±0.2 mm, h = 10± mm), on top of which the tested charge was placed.

After each measurement, the height of the compacted lead cylinder was measured in 4 different points^38^. The brisance of the tested OSM samples was calculated as relative to the brisance of the reference explosive (RDX) charge, but was also expressed as compression in millimetres. The calculations were performed in accordance with Eqs 1 and 2

1 $$\begin{aligned} h_{\hbox {z}}=\frac{h_{\hbox {1}} + h_{\hbox {2}} + h_{\hbox {3}} + h_{\hbox {4}}}{4} \end{aligned}$$

Where:* h*$_{\hbox {z}}$ - average height of the compacted cylinder,* h*$_{\hbox {n}}$ - the height in subsequent measurements.

2 $$\begin{aligned} H_{\hbox {z}}=h_{\hbox {0}} - h_{\hbox {z}} \end{aligned}$$

Where:* H*$_{\hbox {z}}$ - Brisance in mm;* h*$_{\hbox {o}}$ - average height of the cylinder before tests,* h*$_{\hbox {z}}$ - average height of the compacted cylinder.

Brisance was measured only for OSM-K samples (due to OSM-K exhibiting the highest performance out of the tested OSM samples), with each experiment being repeated *n* = 3 times.

### Critical diameter measurement

**Figure 3:**
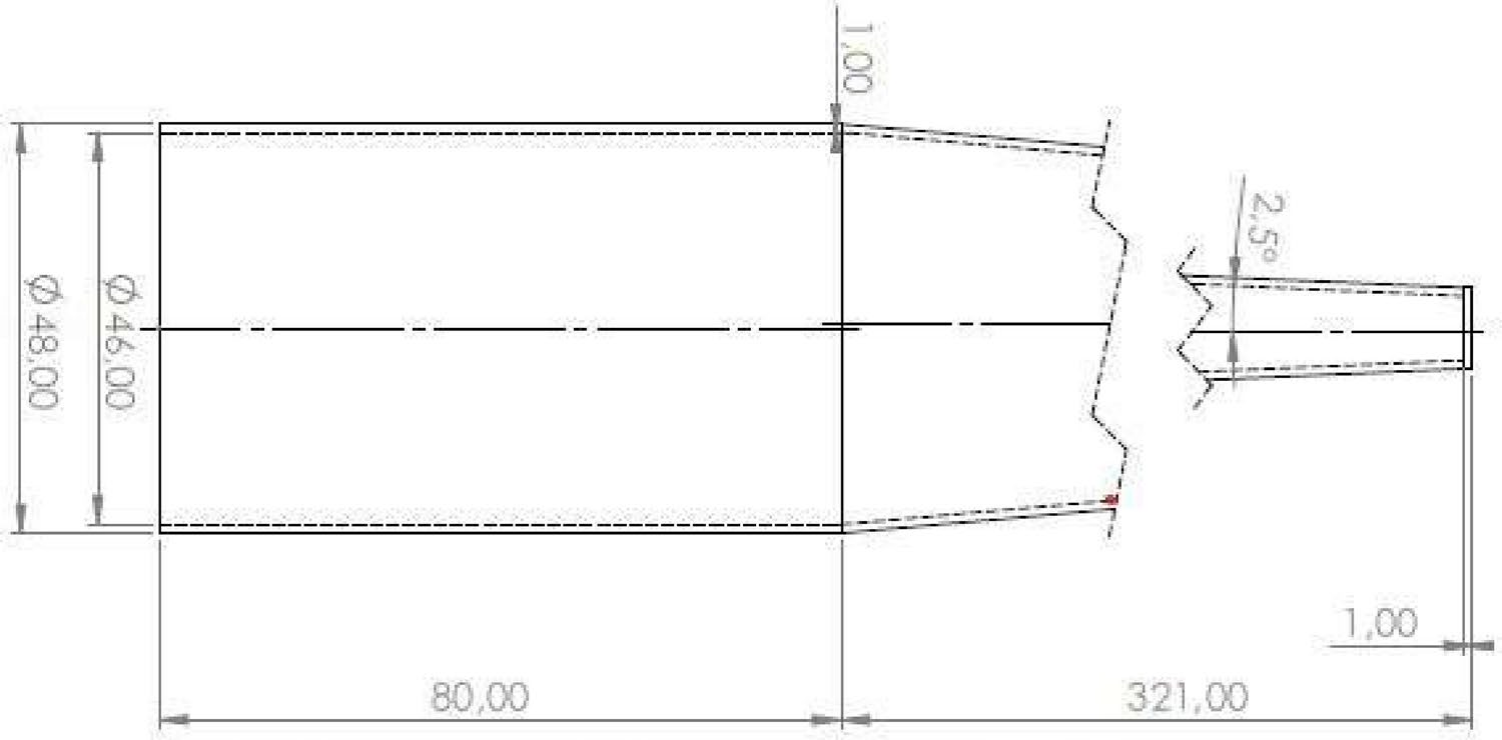
Simplified scheme of the ocnical charge casings used for critical diameter measurements.

Critical diameter was measured for OSM samples loaded into conical charge casings (Fig. 3), which were 3D printed (Prusa Mk3) using PETG polymer (Prusament PETG PRUSA Filament). Before printing, the compatibility of the utilised HP solution and PETG was tested. This involved placing three 1 g pieces of filament inside glass bottles, which were filled with 30 g of the HP solution. The bottles were sealed, weighed and stored for 24 h at room temperature. After this time, the bottles were opened and weighed again – no significant change in mass was recorded.

The cylindrical section of the charges (preceding the conical section, as considered from the point of initiation) was added to minimise the effects of the initiating system on its parameters, by allowing the propagating detonation wave to stabilise in the tested OSM sample. The charges were placed on a S235 steel witness plate (350×150×1 mm) and secured to it with duct tape, in such a way that the entire cone had contact with the plate. After each experiment, the witness plate was collected and cleaned, followed by measurements of the deformation left on the plate using a depth gauge). The critical diameter was equal to the smallest diameter for which detonation occurred.

### Determination of the composition of post-detonation gases

**Figure 4:**
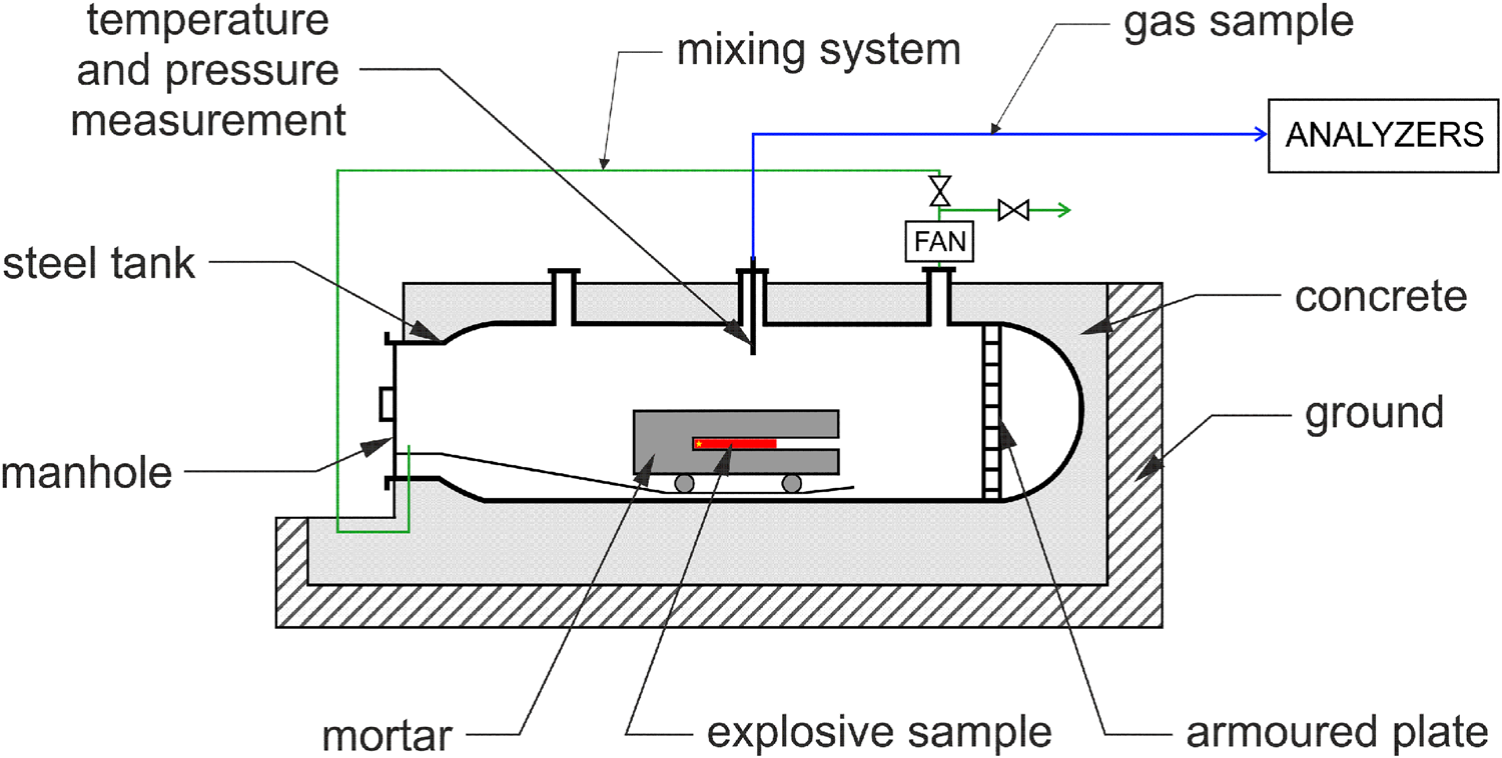
Blasting chamber for post-detonation gases analysis^39^.

All tests were conducted in accordance with the EN 13631-16:2006 standard^7^. OSM-K charges with a mass of 650 g used for these measurements. Glass tubes (50×1.8 mm in diameter, 700 mm long) sealed with 3D printed plugs were used as casings, into which the OSM-K sample was loaded. The plugs were 3D printed from PETG, as was the case for all other 3D printed elements. All charges were initiated with a booster and detonator system. In order to homogenise the mixture of evolved detonation products in the test chamber (Fig. 4), the mixing system was engaged for 3 minutes after each detonation of the tested charges. Chemiluminescent (TOPAZE 32M using a dual chamber for measuring the concentration of nitrogen oxides (NO$_{\hbox {x}}$)) and infrared absorption (MIR 25 for measuring CO and CO$_{\hbox {2}}$ concentrations) detectors were employed to measure the concentrations of toxic gases in the post-detonation gases, utilising a continuous measurement mode (20 min data acquisition). By relating the measured values for a known chamber volume to the mass of the investigated charges, the unit emission of toxic gases was determined.

The obtained results were analysed based on the fact that the concentrations of carbon monoxide (CO) and carbon dioxide (CO$_{\hbox {2}}$) are constant after the initial period of homogenisation of the emitted post-detonation gases. In order to determine the initial concentrations of nitrogen oxides, the changes in their concentrations over the measurement time (20 min) were plotted and the experimental curve was extrapolated to *t* = 0 (corresponding to the detonation event). To calculate the amounts of each gas (QG) under standard conditions (273 K, 760 mmHg) using Eq. 3, the initial concentrations of post-detonation gases (CG) must be known.

3 $$\begin{aligned} QG = \frac{V_{\hbox {ch}}\cdot273}{10^{6} \cdot m\cdot760} \cdot \frac{p_{\hbox {1}}}{T_{\hbox {1}}} \cdot C_{\hbox {G}} [\frac{dm^{3}}{kg}] \end{aligned}$$

Where:

- p$_{\hbox {1}}$ - pressure in the experimental chamber after detonation, mmHg;
- T$_{\hbox {1}}$ - temperature in the experimental chamber after detonation, K;
- V$_{\hbox {ch}}$ - volume of the experimental chamber, dm^3^;
- m - mass of the detonated explosive sample, kg;

## Results and discussion

### Velocity of detonation

**Table 2:** The effect of charge ageing time on the VoD of the OSM samples. VoD values are given in m/s.

Composition	Density g/cm^3^	Ageing time h			
		1	3	6	24
OSM-Ca	1.12	4984	4298	3823	4318
OSM-K	1.06	5106	5003	3838	4278
OSM-Na	1.11	4359	4288	ND	ND

ND - No data.

The highest VoD was observed for OSM-K charges, with OSM-Ca showing very similar properties, even though the density of OSM-K charges is lower than that of the OSM-Ca charges (Table 2). Upon ageing, however, the VoD decreased more rapidly for OSM-Ca than for OSM-K. Both EMs exhibit similar behaviour and trends in the changes of their parameters over time, i.e. VoD decreases for the first 6 h of charge ageing time and is partially restored after 24 h have elapsed.

No data were recorded for OSM-Na charges for ageing times beyond 3 h, due to rapid decomposition of the EM leading to unsealing of the charge covers. Rapid release of gas from the sample surface has also been observed. As such, the samples were deemed to pose a serious threat to the research staff and were disposed via detonation of a 100 g polymer-bonded explosive charge (PMW-14, produced by Mesko S.A.) placed adjacent to them. The significant difference in VoD values recorded after 1 h of ageing in comparison with OSM-Ca and OSM-K samples indicates the occurrence of a faster decomposition process.

During the ageing of the charges, the decomposition of hydrogen peroxide takes place, affecting (lowering) the oxygen balance of the EMs and increasing water content. This translates to decreasing the overall energetic parameters of the formulation. Water is inert in these conditions and plays mostly a texturing role, similar to the case of emulsion EMs^40^. The decreasing oxygen balance of the EM results in incomplete oxidation of the fuels during detonation, as the reaction with gaseous oxygen, evolving in the bulk of the charges, is limited by interfacial contact and rate of gas diffusion. Most likely, the reaction with gaseous oxygen takes place after the reaction zone (i.e. after the CJ plane of the detonation wave). The effect of the described correlation is the decrease of observed VoD values for ageing times of 3 and 6 hours. Due to the fact that the charges were sealed, oxygen, evolved as a result of HP decomposition, remains trapped inside the charges. The population of oxygen bubbles increases during ageing. These bubbles likely play the role of hot-spots, similar to inert gas bubbles intentionally introduced into emulsion EMs during chemical sensitisation^41^, explaining the increase of VoD for the OSM samples observed after ageing the charges for 24 h.

### Pressure parameters

**Figure 5:**
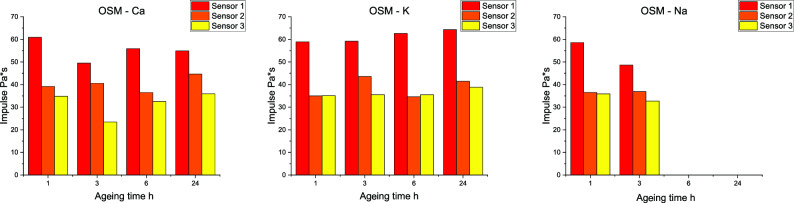
Impulse of tested compositions.

**Figure 6:**
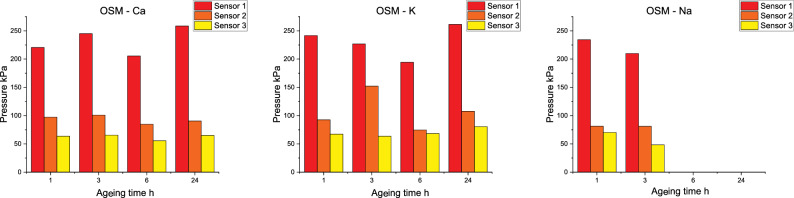
Pressure parameters of tested compositions.

Due to competing influences of decreasing oxygen balance and increasing charge sensitisation, with simultaneous transfer of the decomposition process to the heterogeneous system, the air blast parameters (Figs 5 and 6) were investigated as a function of the distance between each sensor and the studied OSM charge. Analysing OSM-Ca charges for a distance of 1.5 m, an increase in the value of p$_{\hbox {max}}$m correlated with a decrease in the value of the pressure impulse is observed. The p$_{\hbox {max}}$ value increases after 3 h of ageing, decreases slightly after 6 h and reaches its maximum after 24 h of ageing of the charges. In the case of a sensor placed 2.5 m from the charge, the relation is similar, except for the charges aged for 24 h, for which an increase of the maximum overpressure is observed as the impulse increases. For a distance of 2 metres from the centre of detonation, the trends in specific impulse and p$_{\hbox {max}}$ are similar.

**Figure 7:**
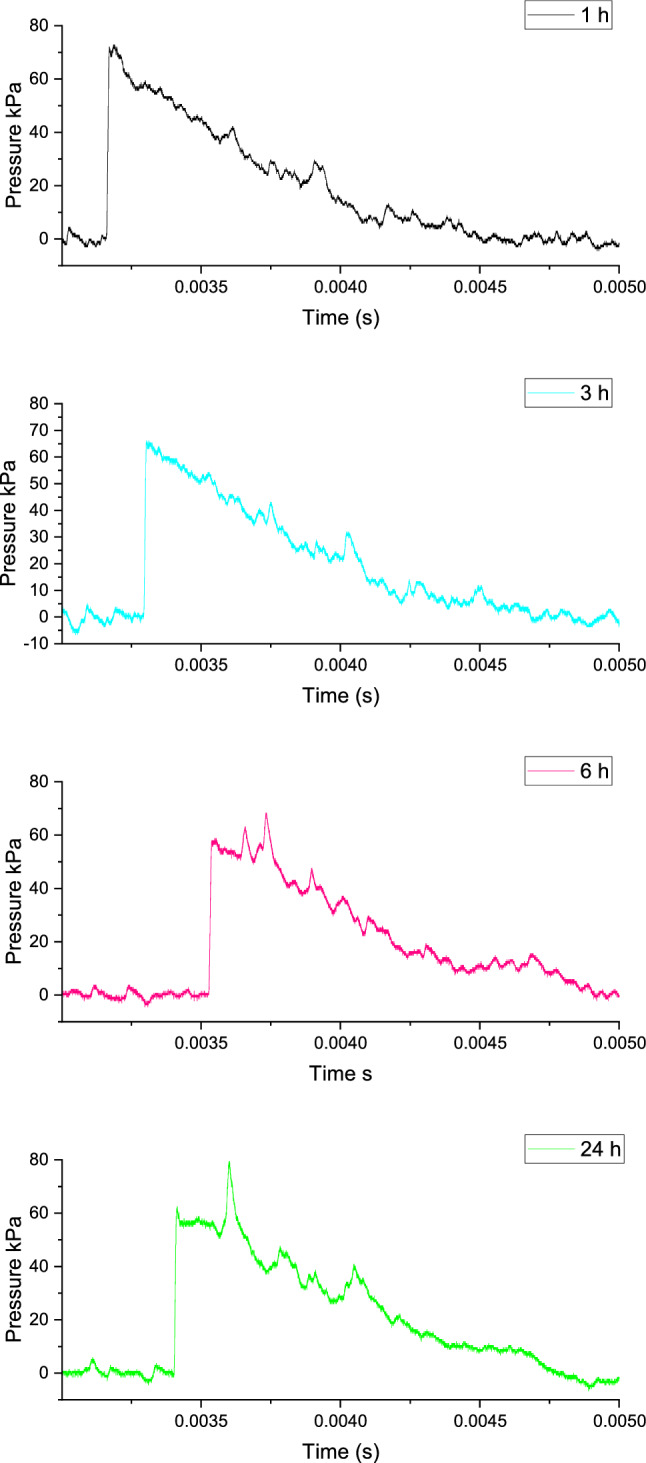
Blast pressure waveforms for OSM-K charges recorded by sensors placed at different distances from the charge, as a function of ageing time.

Analysing the changes in pressure parameters for OSM-K, for the sensor at 1.5 m from the charge, the initial inverse relationship between maximum overpressure and impulse value changes for 24 h of ageing, where an increase in impulse occurs as p$_{\hbox {max}}$ increases. For sensors 2 and 2.5 metres away from the charge, the impulse value increases as the maximum overpressure increases (Fig. 7).

The OSM-Na charges exhibit lower maximum pressure and impulse on the first sensor (1.5 m) and on the third sensor (3 m) for aged sample. Nevertheless, the parameters for the second sensor (2 m) are extremely similar between samples aged for 1 h and for 3 h.

The described relationships are in line with the occurrence decreasing oxygen balance, dilution of the EM with water (forming from HP decay) and the relatively long time scale of the reaction with gaseous oxygen in relation to the time scale of the reactions in the detonation zone. Furthermore, the recorded air blast parameters indicate that decomposition of HP in the OSM-Na charges takes place more rapidly than in the other charges. This leads to lower HP concentrations in OSM-Na than in the other samples and is expected to produce a greater amount of oxygen bubbles in the bulk of the charge. The reaction between the components of the EM and gaseous oxygen is delayed and limited by heterogeneous, interfacial contact. A significant part of the afterburning process occurs during blast wave propagation, which leads to high stability of the observed pressure parameters.

As the charges age, the afterburning of detonation products lengthens, as evidenced by the recorded trends in air blast parameters. The increase in pressure seen on all sensors after 24 h of ageing is likely related to the prevalence of the EM sensitising effect over the desensitising effect of HP decay. This hypothesis is supported by the observed increase in VoD after 24 h of ageing, compared to the VoD recorded after 6 h of ageing.

Depending on the H$_{\hbox {2}}$O$_{\hbox {2}}$/gaseous oxygen ratio, the share of reactions occurring behind the CJ plane changes. This is similar to the afterburning observed for aluminised EMs, in the case of which combustion of larger Al particles^35^ takes place behind the detonation front and during product decay in the blast wave. This hypothesis is consistent with the observed decrease in VoD, as reactions behind the CJ plane do not affect the detonation wave (i.e. have no influence on VoD)^33^.

The differences between the studied charges are due to the different decomposition rates of HP and the differences in properties of the auxiliary oxidising agents. In the work^36^, the decomposition of a series of OSM-type EMs was studied, including those with similar composition, i.e. containing: HP, MgSO$_{\hbox {4}}$, microspheres, guar gum and Na, K or Ca nitrates. A decrease in charge density was recorded, associated with the decomposition of H$_{\hbox {2}}$O$_{\hbox {2}}$ in an open system in a series of Na, K and Ca after 24 h. The observations of this work agree with those recorded for the study discussed here, as evidenced by the high decomposition rate of EMs containing NaNO$_{\hbox {3}}$. At the same time, the time required for the decline of HP concentration by 25% of its initial concentration of H$_{\hbox {2}}$O$_{\hbox {2}}$ increases in the series of K, Na and Ca. This may explain the decrease in pressure parameters of OSM-K and OSM-Na charges after 3 h, relative to OSM-Ca charges.

### Brisance

**Figure 8:**
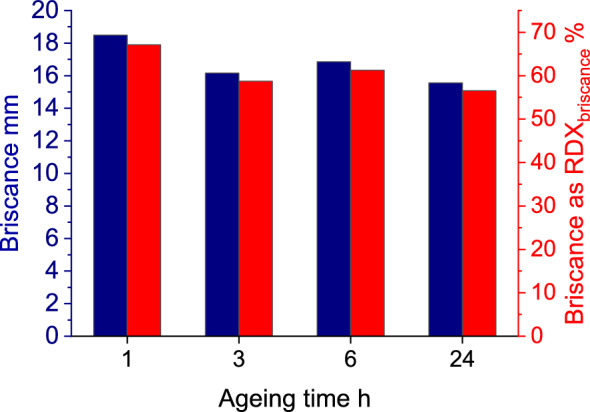
Results of brisance investigations for OSM-K charge using the Hess method, with RDX as the reference.

The results of brisance tests, are presented in Fig. 8.

The tested EMs exhibited high brisance, i.e. >67% of the brisance of the reference EM (RDX). The use of a booster, however, precludes direct comparison of the results with literature^42,43^.

The brisance of EMs is primarily dependent on their VoD and strongly dependent on the detonation pressure. Consequently, the brisance recorded after 1 h of ageing is the highest, as for the charge aged for 1 h, reflecting the loss of VoD upon ageing.

The highest brisance for charges with 1 h ageing time originates from the highest recorded detonation velocity. The subsequent decrease in brisance has similar origin. Nevertheless, the observed brisance increase for 6 h ageing time may be connected with the presence of gaseous decomposition products of the OSM (e.g. oxygen bubbles) within the charges, affecting results as discussed in the case of previous experiments.

### Critical diameter

**Figure 9:**
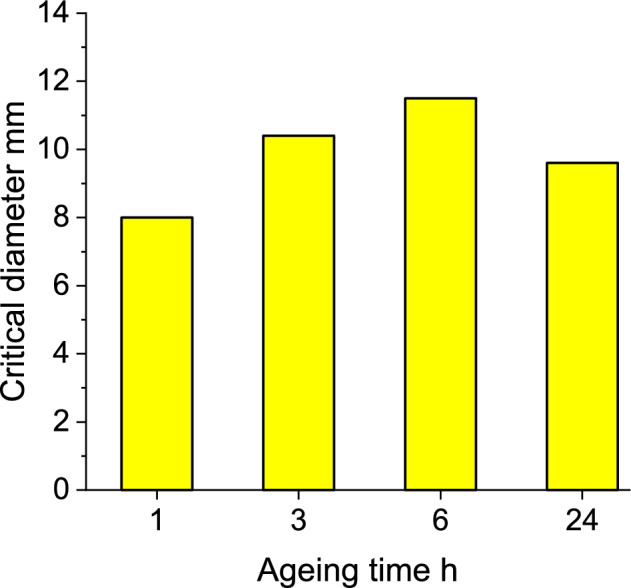
Critical diameter for OSM-K charges.

The changes in the critical diameter of OSM-K over the duration of ageing reflect the observed changes in their VoD (Fig. 9), i.e. the critical diameter increases (evidencing loss of ability to sustain detonation) for the first 6 h of ageing (43.5% increase in comparison with 1 h of ageing), followed by a decrease in the case of charges aged for 24 h (20% increase in comparison with 1 h of ageing). The results indicate that the postulate of competition between HP decomposition (leading to lower content of active components) and increase of hot-spot population (leading to higher sensitivity to initiation and facilitating the detonation process) is most likely correct. Importantly, even after 24 h of ageing, the critical diameter of the OSM-type EMs is remarkably low, especially so for a non-ideal EM, as it less than half of the critical diameter of typical ANFO charges (usually, above 20 mm^44^) or even commercial emulsion EMs (e.g. 34 mm for Emulinit 7^45^).

### Composition of post-detonation gases

**Figure 10:**
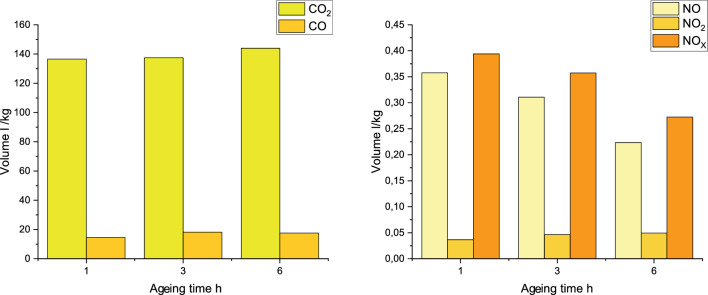
Concentrations of toxic fumes after detonation of OSM-K explosives.

Ageing of OSM-K results in increasing the CO and CO$_{\hbox {2}}$ concentrations and decreasing the NO$_{\hbox {x}}$ concentrations in post-detonation gases (Fig. 10, Table 3). This is largely tied to the decay of HP and is in line with what is expected for charges with decreasing oxygen balance values. The decreasing concentrations of the active oxidising agent (HP) and its replacement with a less active oxidising agent (gaseous oxygen) result in a decrease in the concentration of nitrogen oxides at the expense of an increase in the concentrations of carbon oxides, including the more dangerous CO. Roughly constant oxygen balance in whole charge was confirmed by weight of charges measurement after preparation and right before detonation. In each case, the difference in mass was no larger than 0.2 wt.%, which confirms trapping of gaseous oxygen inside charges. The results of the measurements also confirm the hypothesis of limited reactivity of the gaseous oxygen present in the system. In light of this, the oxygen balance during ageing should be calculated based on the HP concentration at the time and largely disregarding the presence of gaseous oxygen.

The investigated OSMs showed complex behaviour compared to commercially available and popular EMs. With reference to the literature data presented, the CO$_{\hbox {2}}$ concentration is higher than in most emulsion EMs, but lower than in the case of dynamites and other ammonium nitrate-based EMs. The CO concentration is very high, even in relation to dynamites. Only the emulsion EMs, described as MWE 1-3, exhibited a higher content of CO in the post-detonation gases.

Conversely, the emission of nitrogen oxides from the OSM EMs is extremely low - the lowest among all the listed EMs, regardless of OSM charge ageing time. The NO concentration is similar to values typical for modern emulsion EMs, such as BK-1 or BK-2. Moreover, the NO$_{\hbox {x}}$ concentration is significantly lower than currently widely used EMs, such as Emulinit 8L, dynamites or typical ammonium nitrate-based EMs.

Summarising, while the OSM-K charges exhibited moderately high emissions of carbon oxides, they feature the lowest concentrations of nitrogen oxides among the most common EMs used for civilian purposes. Nevertheless, the general relative toxicity, expressed as a CO + 6.5 · NO$_{\hbox {x}}$, is moderate. Additionally, the results were slightly disrupted by toxic gases coming from the decomposition of the booster and the PETG plugs.

**Table 3:** Toxic gases concentration in detonation products of different explosives.

Explosive	CO$_{\hbox {2}}$, dm^3^/kg	CO, dm^3^/kg	NO, dm^3^/kg	NO$_{\hbox {2}}$, dm^3^/kg	NO$_{\hbox {x}}$, dm^3^/kg	RT$_{\hbox {*}}$	Ref.
OSM-K-1h	136.6	14.6	0.36	0.04	0.39	17.13	This work
OSM-K-3h	137.5	18.2	0.31	0.05	0.36	20.54	This work
OSM-K-6h	144	17.6	0.22	0.05	0.27	19.36	This work
Emulnit 8L	114.8	4.11	0.51	0.04	0.55	7.69^**^	^39^
BK-1	117.1	2.51	0.29	0.04	0.33	4.66^**^	^39^
BK-2	115.3	3.45	0.28	0.03	0.31	5.47^**^	^39^
Ammonite 1	145.15	5.87	3.58	1.15	4.74	36.65	^7^
Ammonite 2	110.22	2.63	0.77	0.56	1.22	10.56	^7^
Dynamite 1	167.45	5.93	0.92	0.07	0.99	12.38	^7^
Dynamite 2	181.6	4.37	2.61	4.5	0.64	8.53	^7^
Dynamite 3	185.12	4.58	1.89	0.11	2	17.59	^7^
Dynamite 4	171.53	1.56	5.49	0.46	5.96	40.31	^7^
MWE 1	109.34	21.85	0.62	0.06	0.68	26.28	^7^
MWE 2	123.72	21.43	1.09	0.06	1.15	28.90	^7^
MWE 3	105.26	21.43	0.38	0.02	0.4	24.03	^7^
Methanite 1	91.85	9.29	3.69	0.16	3.86	34.35	^7^
ANFO	94,19	13,91	1,53	0,11	1,64	24,57	^46^

ND - No data.

$_{\hbox {*}}$ RT - Relative Toxicity expressed as: CO + 6.5 · NO$_{\hbox {x}}$

^**^ Calculated according to formula above. NO$_{\hbox {x}}$ concentration calculated as a sum of NO$_{\hbox {2}}$ and NO concentration.

## Discussion

The spontaneous decay of HP in OSM-type EM charges strongly influences their detonation parameters. The detonation parameters of all tested OSM samples decreased upon ageing times beyond 1 h, even though that decrease was not linear, due to the beneficial effect of OSM sensitisation by oxygen gas bubbles.

The decay of HP led to an interesting, complex and multi-step energetic process. Firstly, the main detonation process is strongly influence by the changes of component concentrations, the decreasing oxygen balance and sensitisation by O$_{\hbox {2}}$ gas bubbles trapped in the bulk of the OSM charges. These phenomena translate to the discussed changes in VoD, brisance and critical diameter. Secondly, the oxygen evolving from HP decay is not lost from the charges, but reacts after the CJ plane, inducing afterburning. The afterburning does not affect the propagating detonation wave, but contributes to augmenting the air blast parameters (maximum overpressure and impulse) at relatively high levels, granting the OSM-type EMs high utility in civilian blasting operations. This feature shows similarity to the features of thermobaric and enhanced blast EMs, such as aluminised EMs. Moreover, the texturing role of water in this case should not be disregarded, as it strongly influences the propagation of the detonation wave.

In terms of the effect of ageing on the performance of OSM-type EMs, even after 24 h of ageing, the charges show detonation parameters comparable if not exceeding that of currently used emulsion EMs, such as Emulinit 7L (3900 m/s), Emulinit 8L (3800 m/s)^45^. The VoD, in particular, remains high after prolonged ageing, being comparable even with modern emulsion EMs, such as BK-1 (4637 m/s) and BK-2 (5033 m/s)^39^, and nitric acid ester-based EMs, such as SB-520 (5032-5203 m/s) or DSB-20 (5183-5185 m/s)^32^.

Interestingly, the post-detonation gases show moderate toxicity. The concentration of NO$_{\hbox {x}}$ is extremely low for OSM-K-1h charges and decreases even further upon charge ageing. Conversely, the post-detonation gases contain an appreciable share of carbon monoxide (CO). The emission of CO can be greatly limited during the optimisation of the OSM formulations, based on these results. That being said, even without optimisation, the relative toxicity of OSM-K charges is lower than that of AN based EMs and many emulsion EMs or dynamites (Table 3).

The increasing share of CO upon OSM charge ageing indicates that the effective oxygen balance of the EM decreases over time. This appears not to match the fact that oxygen bubbles, originating from the decomposition of HP, remain trapped within the bulk of the OSM charges, as this “oxygen bubble trapping” would prevent any significant changes to the oxygen balance of OSM. The contact of these oxygen bubbles with the fuel is, however, limited and decreases with increasing ageing time (i.e. with increasing average diameter of these bubbles). Consequently, the oxygen contained within these bubbles cannot react with the fuel upon detonation, even if these bubbles remain capable of serving as hot-spots.

In light of the above, the OSM EMs can be considered to completely mitigate the risk of environmental pollution with non-decomposed nitric acid esters, which is present e.g. in the case of using dynamites^32^. The self-sensitising mechanism of OSM ageing indicates that this class of EMs can be developed to undergo timed self-arming after being loaded into blast holes, without the need of introducing any sensitising additives and without the need of manipulation by personnel. This mechanism also resolves the issue of the sensitising process causing the emission of toxic compounds.

## Conclusions

Among the investigated OSM formulations, OSM-K exhibited the most favourable performance. The formulation showed high stability of detonation parameters over ageing, especially in the case of VoD and pressure parameters. The observed high VoD is similar or even higher than that of many EMs that are currently widely used in civilian blasting operations. The critical diameter of OSM-K is extremely low (less than half of the >20 mm critical diameter values typically reported for ANFO) for a non-ideal EM and is beyond sufficient for civilian applications (e.g. mining industry) to consider application of this material in mining industry. The brisance of OSM-K is approx. 67% of the brisance of RDX (model EM), being comparable or exceeding the brisance of most EMs used in civilian applications.

The NaNO$_{\hbox {3}}$-based OSM showed the worst performance and the fastest HP decomposition rate. This formulation should be completely rejected from further tests, due to low stability and generation of hazardous situations. In turn, OSM-Ca showed average parameters even despite exhibiting the highest density from among the investigated OSM formulations.

The tested EMs demonstrated an interesting mechanism affecting their parameters over time. Even though the the active oxidising agent (HP) content decreases due to its spontaneous decay, the increasing number of hot-spots maintains the performance of the EM. The delayed reaction between partially oxidised detonation products and gaseous oxygen, evolving as bubbles in the bulk of the charges, leads to high stability of blast wave parameters. This stability is highly desirable, as the air blast parameters are a strong measure of the ability of the EM to perform mechanical work.

The performance of the investigated OSMs was strongly dependent on their ageing time, as expected on the grounds of our previous works. The HP decomposition rate can be controlled and optimised, in order to develop EMs that undergo both timed self-arming and timed self-deactivation. Moreover, by controlling the charge ageing time (i.e. delay between preparation of EM charges and performing the blasting operation), the energetic performance of the OSMs can be attenuated as needed, allowing a single EM formulation to be used in a variety of different conditions.

OSM-K charges exhibited mediocre toxicity. The benefits coming from extremely low NO$_{\hbox {x}}$ concentration are partially reduced by a rather high concentration of carbon monoxide, but this drawback can be greatly limited, if not resolved entirely, through optimising the OSM formulations based on data reported in this work. Moreover, even prior to optimisation, the OSM formulations contain no toxic components, eliminating both the risk of environmental contamination and the risk of personnel exposure. Instead, the main oxidising agent, i.e. HP, is produced in environmentally-friendly process. Similarly, the main fuel, i.e. glycerine, is a by-product of many industrial processes, such as the transesterification of oils, providing a stark contrast to the typical profile of EM components.

The self-sensitising process, may be further tailored, so as to eliminate the need for external sensitising agents (chemical or physical agents), eliminating their impact on the environment and making the OSM-type EMs even “greener”.

The reported energetic parameters of OSM show that these EMs can find an application as a mining blasting agent, especially due to their high ability to perform mechanical work and high VoD. The toxicity of OSM detonation products can likely be greatly decreased through optimisation based on the first data about the composition of their post-detonation gases reported herein. This optimisation will likely require removing the auxiliary oxidising agent (nitrates) and developing formulations that maintain a slightly positive oxygen balance over their window of use.

## Supplementary Information

Supplementary Information.

## Data Availability

The data that support the findings of this study are available on reasonable request from the authors.
