# Supplementary material for: Comprehensive study on impact of hydrogen peroxide decomposition on the crucial parameters of OSM-type energetic materials
Source: Sci Rep. 2024 Jun 18;14:14093. doi: 10.1038/s41598-024-64974-w (PMC11637108; doi:10.1038/s41598-024-64974-w)
Supplement: Supplementary file 1 — Supplementary Information. [file 41598_2024_64974_MOESM1_ESM.pdf]

A Supplementary materials

Table A1. Materials used in this work.

Chemical (code)	Purity grade	Source
Sodium nitrate ($NaNO_3$)	>99 %	Chempur (Piekary Śląskie, Poland)
Potassium nitrate (KNO_3)	>99 %	Chempur (Piekary Śląskie, Poland)
Calcium nitrate tetrahydrate ($Ca(NO_3)_2 \times 4H_2O$)*	>99 %	Chempur (Piekary Śląskie, Poland)
Hydrogen peroxide 60 wt. % solution (HP)	analytical	Chempur (Piekary Śląskie, Poland)
Glycerine	>99.5 %	TechlandLab (Tarnobrzeg, Poland)
Guar gum S.C.-406 (GG)	>99 %	Meyhall Chemical AG (Kreuzlingen, Switzerland
Glass microspheres type K-015 (MS)	n/a	3M (Saint Paul, MN, USA)

*The reagent was dried in 373K for 24 h before use, to remove the hydration water

Table A2. Detonation velocity reported for explosives containing concentrated HP that have been described in literature.

Fuels	Auxiliary substances	D [$m \cdot s^{-1}$]	Ref.
GC	AN, gMS	5400–5700	28
GC, Al	AN, gMS	3600–5500	35
GC	gMS / pMS / gas bubbles	3000–5500	29
GC	gMS	2600–5100	30
GC, Al	AN, gMS	4400–5200	31

GC - Propano-1,2,3-triol AN - Ammonium nitrate(V)
gMS - Glass microspheres pMS - Polymer microspheres

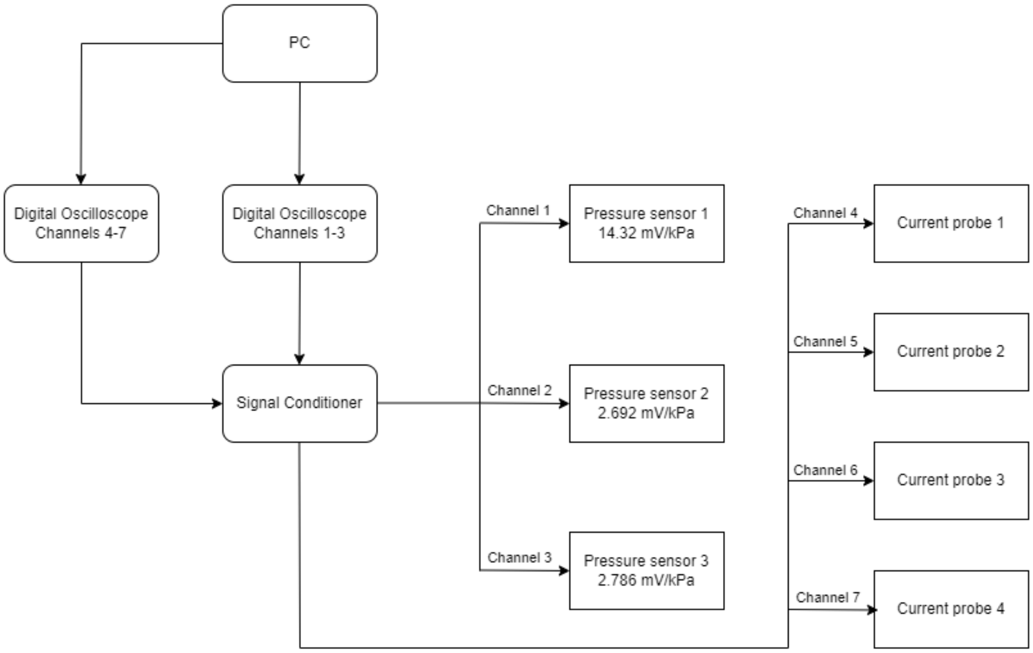

Figure A1. Block diagram of the experimental set-up employed for conducting air blast parameters and velocity of detonation.
